# Preliminary Characterization of Glyceride Oil Content and Tocopherol Composition in Seeds from Selected Wild Plant Species of the Bulgarian Flora

**DOI:** 10.3390/molecules30142893

**Published:** 2025-07-08

**Authors:** Zhana Petkova, Ginka Antova, Hristina Kulina, Olga Teneva, Maria Angelova-Romova

**Affiliations:** 1 Department of Chemical Technology, Faculty of Chemistry, University of Plovdiv “Paisii Hilendarski”, 24 Tzar Asen Street, 4000 Plovdiv, Bulgaria; zhanapetkova@uni-plovdiv.bg (Z.P.); olga@uni-plovdiv.bg (O.T.); maioan@uni-plovdiv.bg (M.A.-R.); 2 Department of Mathematical Analysis, Faculty of Mathematics and Informatics, University of Plovdiv “Paisii Hilendarski”, 24 Tzar Asen Street, 4000 Plovdiv, Bulgaria; kulina@uni-plovdiv.bg

**Keywords:** Bulgarian wild flora, tocopherols, glyceride oils, hierarchical cluster analysis

## Abstract

Tocopherols, due to their antioxidant properties, are valuable compounds in the food and pharmaceutical industries. The present study compares the glyceride oil content and tocopherol profile of seeds of 49 plant species from 39 families of Bulgarian wild flora to identify their potential industrial and nutritional applications. The oils were extracted using the Soxhlet apparatus, and the tocopherol and tocotrienol composition was established by high-performance liquid chromatography (HPLC). Hierarchical cluster analysis was performed to group the plants based on their tocopherol profile. The results show high variability in the content of glyceride oils (ranging from 0.5% to 40.6%) and tocopherol profiles among species, even among plants within the same family. Four clusters were identified, each of which was characterized by the dominance of one of the tocopherol or tocotrienol isomers, i.e., α-tocopherol or γ-tocopherol, and reflected the chemical diversity of the examined plants. The statistical analysis confirmed that tocopherols and tocotrienols are significant factors influencing cluster grouping. The results reflect natural variability among species grown under field conditions, influenced by both genetic and environmental factors. This study provides valuable preliminary information for identifying wild species with promising tocopherol profiles for future functional research.

## 1. Introduction

Tocopherols are organic compounds with vitamin E activity, acting as lipid-soluble antioxidants. Found in glyceride oils from nuts and seeds, tocopherols and tocotrienols belong to the tocochromanols group [1]. The structure includes a chromanol ring and a phytyl tail for lipid solubility. Each consists of four homologues: α-, β-, γ-, and δ-, differing in methyl group positions on the ring [2,3]. While α-tocopherol, the most biologically active, is common in European diets, γ-tocopherol is prevalent in American diets [1]. Synthesized by photosynthetic organisms like plants and cyanobacteria, tocopherols are primarily sourced from vegetable oils, nuts, and seeds. Their absorption depends on dietary lipids, other vitamins, and individual metabolism, with α-tocopherol preferentially secreted into the bloodstream after liver processing [1]. As potent antioxidants, tocopherols protect cell membranes from oxidative damage, prevent lipid peroxidation [2], and reduce the risks of cardiovascular and neurodegenerative diseases. They exhibit anti-inflammatory properties and are used in the food industry to prevent lipid oxidation and maintain product quality [2]. Nevertheless, ongoing research continues to uncover new aspects of their bioactivity and potential therapeutic applications.

Bulgaria is renowned for its rich biodiversity and diverse plant species. The country’s varied topography and climate create a favorable environment for various plant species. Bulgaria has a temperate continental climate, with hot summers and cold winters. Diverse geographical features, such as the Rhodope Mountains, fertile plains, and river valleys, contribute to this floral diversity [4].

Many of Bulgaria’s wild flora plants and their seeds find application as spices and ingredients in drugs in nontraditional medicine [5,6]. The content and composition of the lipids of these plants are of considerable scientific and practical interest because of the presence of biologically active substances: tocopherols, sterols, and phospholipids. The efforts to establish the tocopherol composition worldwide focus on the most common edible seeds and nuts rich in glyceride oils. Total tocopherol content of the most used vegetable oils varied between 60 mg/kg (in Babassu oil) and 1300–3720 mg/kg in arachis, cottonseed, palm, rapeseed, rice bran, soybean, and maize oils [7]. On the other hand, there is limited information about the content of these lipid-soluble bioactive compounds in wild plant species. Nehdi et al. [8] reported that *Allium ampeloprasum* seed oil contained two major tocols, γ- and δ-tocotrienols, in quantities of 79.56 and 52.08 mg/100 g oil. The seed oil from *Lannea velutina* from the family Anacardiaceae had γ-tocopherol (36.1 mg/100 g), and the other detected components in much lower amounts were α- and δ-tocopherol [9]. Velasco and Goffman [10] examined the lipid composition of 45 accessions from the family Boraginaceae, including their tocopherol composition. They established that total tocopherol content was the highest in *Anchusa arvensis* (L.) M. Bieb. (1765 mg/kg) and *A. officinalis* L. (1729 mg/kg), and lowest in *Omphalodes linifolia* (L.) Moench (91 mg/kg) and *Heliotropium arborescens* L. (131 mg/kg). Three tocopherol isomers were detected in all samples: α-, γ-, and δ-tocopherol. The two plants with the presence of only α-tocopherol were *L. longiflora* (Benth.) Baill. and *Solenanthus apenninus* (L.) Fisch. & Mey. The six species that possessed only γ-tocopherol were *H. arborescens*, *Omphalodes linifolia* (L.) Moench, *Lithospermum officinale* L., *Onosma arenarium* W. & K., *Myosotis alpestris* F.W. Schmidt, and *M. sylvatica* (Ehrh.) Hoffm., and the rest of the samples had different ratios of the detected tocopherols. The same authors reported that *Borago officinalis* L. and *Pulmonaria mollis* Wulfen contained the highest amount of δ-tocopherol (88.1–90.4 and 38.1% of the total tocopherols, respectively). Ogrodowska et al. [11] and Petkova et al. [12] examined the tocopherol composition of amaranth seeds (family Amaranthaceae) from Poland, India, and Turkey and established that the major components were β- and δ-tocopherols. The total content was 10.61 mg/100 g in the Polish samples, but much higher in the oils from the Indian and Turkish species: 1015–1060 mg/kg. Other seeds that were rich sources of tocopherols, especially β-tocopherol, were from the plant *Zizyphus lotus* from the Rhamnaceae family [13]. Their total tocopherol content was found to be 141.07 mg/100 g, and that of the β-isomer was 130.47 mg/100 g. β-Tocopherol (2.19 g/kg) was also the major component of the wild plant *Cistanche phelypaea* (Orobanchaceae), followed by α-tocopherol (0.75 g/kg), and there were negligible amounts of δ- and γ-tocopherol (0.27 and 0.14 g/kg) [14]. Tocotrienols are rarely found in nature, but there are some previous studies that show that tocotrienol is present in higher amounts in different parts of wild *Hypericum perforatum* L., including the leaves, flowers, stems, flower buds, dead petals, and unripe seed pods [15].

Despite the previous studies on the tocopherol composition of some wild plant species, most representatives of Bulgaria’s wild flora have not been investigated so far regarding the content and composition of the lipids in the seeds, especially the tocopherols. It is also interesting to seek a connection between the content of lipids in the seeds and the content of tocopherols in the lipids and the seeds of those plants.

Given the biochemical importance of tocopherols and the limited research on their composition in wild plant species, we hypothesize that the tocopherol and tocotrienol profiles of seed oils from Bulgarian wild flora exhibit significant variability that is not strictly dependent on plant family or ripening time. We further propose that certain wild species may serve as valuable sources of specific tocopherol isomers with potential industrial applications in the future. Therefore, the aim of this study is to characterize the glyceride oil content and tocopherol composition in the seeds of 49 plant species from the Bulgarian wild flora using high-performance liquid chromatography (HPLC) and to explore biochemical patterns among the species through hierarchical cluster analysis. The results are intended to serve as a basis for identifying plant species with promising lipid-soluble antioxidant profiles for further functional and application-focused research.

## 2. Results and Discussion

The oil content, total tocopherol content, and individual tocopherol composition were determined in seeds from the following plant species: Alliaceae—*Allium sardoum* Moris; Anacardiaceae—*Cotinus coggygria* Scop. and *Rhus typhina* L.; Asparagaceae—*Asparagus officinalis* L.; Boraginaceae—*Nonnea pallens* Petrovič; Amaranthaceae—*Chenopodium album* L.; Chenopodiaceae—*Beta trigyna* Waldst. & Kit. and *Salsola ruthenica* Iljin.; Cistaceae—*Cistus creticus* L.; Convolvulaceae—*Calystegia sepium* (L.) R. Br.; Cucurbitaceae—*Bryonia alba* L. and *Bryonia cretica* L.; Cuscutaceae—*Cuscuta monogyna* Wahl.; Cyperaceae—*Cyperus esculentus* L.; Dioscaraceae—*Dioscorea communis* (L.) Caddick & Wilkin; Dipsaceae—*Cephalaria laevigata* W.K.Schrad. and *Cephalaria transsilvanica* Schrad.; Euphorbiaceae—*Euphorbia helioscopia* L.; Ginkgoaceae—*Ginkgo biloba* L.; Geraniaceae—*Erodium cicutarium* (L.) L’Hér.; Juncaceae—*Juncus compressus* Jacq.; Liliaceae—*Asphodeline lutea* Rchb.; Santalaceae—*Viscum album* L.; Lythraceae—*Lythrum salicaria* L.; Malvaceae—*Althaea heldreichii* Boiss., *Althaea officinalis* L., and *Malva silvestris* L.; Moraceae—*Broussonetia papyrifera* (L.) Vent.; Onagraceae—*Oenothera biennis* L.; Plumbaginaceae—*Plumbago europaea* L.; Portulaceae—*Portulaca oleracea* L.; Resedaceae—*Reseda luteola* L. and *Reseda lutea* L.; Rhamnaceae—*Paliurus spina-christi* Mill.; Rutaceae—*Haplophyllum suaveolens* (DC.) G.Don and *Ruta graveolens* L.; Sapindaceae—*Koelreuteria paniculata* Laxm.; Scrophulariaceae—*Digitalis lanata* Ehrh. and *Verbascum lychnitis* L.; Simarubaceae—*Ailanthus altissima* (Mill.) Swingle; Solanaceae—*Hyoscyamus niger* L. and *Nicotiana tabacum* L.; Sparganiaceae—*Sparganium erectum* L.; Trapaceae—*Trapa natans* L.; Ulmaceae—*Celtis australis* L.; Urticaceae—*Urtica dioica* L.; Verbenaceae—*Vitex agnus-castus* L.; Violaceae—*Viola arvensis* Murray; and Zygophyllaceae—*Zygophyllum fabago* L.

The contents of glyceride oil in the seeds and of tocopherols in the oils and the seeds, respectively, are presented in Table 1.

The glyceride oil content of the examined plant seeds varied in a broad range from 0.5% in *T. natans* to 40.6% in *V. arvensis*. Rich in glyceride oil (over 20.0%) were the seeds of *C. esculentus*—22.5%, *C. transsilvanica*—21.1%, *A. lutea*—21.4%, *B. papyrifera*—26.0%, *H. niger*—21.4%, *H. niger*—23.4%, *N. tabacum*—34.5%, *C. australis*—21.0%, *U. dioica*—22.7% and *V. arvensis*—40.6%. These results are similar to the glyceride oil content of commonly used oily seeds such as soybean (16–21%) and rapeseed (39–42%), but lower than that of sunflower seeds (45–50%) [16]. The present results were also in agreement with the glyceride oil content of some non-traditional oilseed plants such as black cumin (*N. sativa* L., 26–34%), evening primrose (*O. biennis* L., 20–30%), hemp (*C. sativa* L., 25–35%), and milk thistle (*S. marianum* (L.) Gaertn, 20–30%) [17].

Fourteen of the examined plant species had lipid content between 10 and 20%, with the highest amount among them being in *B. alba* (19.3%), *C. laevigata* (18.8%), *N. pallens* (18.1%), *B. cretica* (17.5%), *P. spina-christi* (17.0%), and *A. altissima* (17.0%). Similar quantities of glyceride oil were observed in seeds from Mexican cotton (*G. hirsutum*, 19.6%) and purple allamanda (*A. blanchetti*, 13.4%) [18].

The rest of the examined plant species possessed total lipids in amounts below 10%; those with the lowest quantity being the seeds from *T. natans* (0.5%), *S. erectum* (0.7%), *A. sardoum* (1.2%), *J. compressus* (1.2%), *A. officinalis* (1.4%), and *B. trigyna* (1.7%).

The content of tocopherols in the oils varied greatly from oils with a low content (below 200 mg/kg) to oils with a high content of tocopherols (above 800 mg/kg). The richest in tocopherols were the oils of *A. sardoum* seeds 8066.5 mg/kg, *V. lychnitis*—3378.3 mg/kg, *C. monogyna*—2858.2 mg/kg, *C. coggygria*—1763.9 mg/kg, *B. papyrifera*—1494.8 mg/kg, *K. paniculata*—1099.0 mg/kg, *V. album*—1077.4 mg/kg, *E. cicutarium*—928.3 mg/kg, and *C. sepium*—829.2 mg/kg. In general, the seeds from the same plants were also the richest in tocopherols (above 100 mg/kg in the seeds). The highest content of total tocopherols was observed in the following plant seeds: *B. papyrifera*—388.3 mg/kg, *V. lychnitis*—193.0 mg/kg, *C. monogyna*—162.7 mg/kg, *C. australis*—152.7 mg/kg, *C. coggygria*—148.2 mg/kg, and *E. cicutarium*—111.4 mg/kg. The observed variability in tocopherol content among species likely reflects inherent genetic differences and the influence of natural environmental growing conditions.

The total tocopherol content that was present in high amounts in the lipids from the examined wild plants (from 1077.4 mg/kg in *V. album* to 3378.3 mg/kg in *V. lychnitis*) was similar to that of the most common edible oils from peanut, cottonseed, palm, rapeseed, rice bran, soybean, and maize oils (1300–3720 mg/kg) [7]. On the other hand, the amount of tocopherols found in *A. sardoum* was much higher than in the previous studies on widespread vegetable oils.

The qualitative and quantitative composition of the tocopherol fraction is presented in Table 2.

The data reveal a diverse qualitative composition among the investigated plant oils, with a predominance of saturated derivatives, specifically tocopherols. In terms of α-tocopherol, the highest content was observed in 17 oils, with 6 of these oils exhibiting levels above 80.0%. These include *C. laevigata*—81.1%, *B. cretica*—85.5%, *P. spina-christi*—87.7%, *J. compressus*—91.1%, *A. altissima*—96.4%, and *A. heldreichii*—98.0%. A notable presence of β-tocopherol was only detected in the oil of *U. dioica* at 55.2%. γ-tocopherol was identified at levels higher than 50.0% in 9 oils, with the highest concentrations observed in *C. album*—83.2%, *C. sepium*—85.1%, *E. cicutarium*—84.2%, *P. oleracea*—98.8%, *R. lutea*—82.8%, *N. pallens*—59.1%, *V. album*—66.0%, *H. suaveolens*—98.5% and *N. tabacum*—52.0%. δ-Tocopherol was predominant in 10 oils, with the highest amounts found in *D. communis* at 91.7%, *S. erectum* at 84.6% and *C. australis* at 98.7%. Higher amounts of over 50% of the same tocopherol isomer were detected in *B. trigyna*—52.7%, *C. monogyna*—50.3%, *A. lutea*—72.8%, *B. papyrifera*—74.1%, *K. paniculata*—75.0%, and *T. natans*—55.4%.

Among the tocotrienols, α-tocotrienol was detected in significant quantities in the oils of only three plant species: *R. typhina*—54.7%, *C. transsilvanica*—63.2%, and *M. silvestris*—82.0%. β-tocotrienol was detected in negligible amounts in *R. graveolens*—5.8% and *V. agnus-castus*—10.0%. γ-tocotrienol was detected in a high concentration of 13.1% in *V. agnus-castus*, and δ-tocotrienol was identified in negligible amounts in four plant seeds: *C. monogyna*, *E. cicutarium*, *H. suaveolens*, and *K. paniculata*.

The high levels of α-tocopherol in *C. laevigata*, *B. cretica*, *P. spina-christi*, *J. compressus*, *A. altissima,* and *A. heldreichii* (from 81.1 to 98.0%) were similar to the data of *L. longiflora* (Benth.) Baill. and *Solenanthus apenninus* (L.) Fisch. & Mey., which possessed only this tocopherol isomer [10]. *N. pallens* from the family Boraginaceae had a similar composition to *Lappula squarrosa* (Retz.) Dumort, where γ-tocopherol was the main component (92.7%), followed by δ-tocopherol (7.3%) [10]. The composition of *U. dioica* was similar to that of amaranth seeds (family Amaranthaceae) from Poland, India, and Turkey, the major components being β- and δ-tocopherols as well [11,12]. The major component in *C. album*, *P. oleracea*, *R. lutea,* and *H. suaveolens* was γ-tocopherol, such as the seed oil from *L. velutina,* but the other components (α- and δ-tocopherol) were detected in much lower amounts [9]. The examined *C. australis* and *S. erectum,* which were rich in δ-tocopherol, had similar compositions to *Allium ampeloprasum* seed oil, in which the content of δ-tocotrienol was 52.08 mg/100 g [8]. The tocotrienol content was high in ten plant species, comparable to the elevated levels found in various parts of the wild *H. perforatum*, where tocotrienols are also abundantly present [15].

The variability in tocopherol composition among oils from plants within the same botanical family suggests that tocopherol profiles are not reliable indicators for plant species identification within families. This variability underscores the complexity and diversity of tocopherol distribution among different plant species and highlights the need for comprehensive profiling when assessing the potential applications of plant-derived oils in various industries. The use of triplicate composite samples in this preliminary phytochemical survey is a widely accepted practice for initial screenings, providing sufficient statistical reliability for identifying broad patterns and variability among species.

For our study, we applied a hierarchical agglomerative algorithm with average-link clustering. Distance was measured using Euclidean distance. Initially, we applied the analysis with all the examined variables—oil_content, TTC, α-tocopherol, α-tocotrienol, β-tocopherol, β-tocotrienol, γ-tocopherol, γ-tocotrienol, δ-tocopherol, and δ-tocotrienol. The conducted ANOVA shows that the variables oil_content (*p* = 0.575), TTC (*p* = 0.301), β-tocotrienol (*p* = 0.503), γ-tocotrienol (*p* = 0.629), δ-tocopherol (*p* = 0.541), and δ-tocotrienol (*p* = 0.828) do not have a significant influence on the separation of the cluster groups. The ANOVA results showed that the influences of α-tocopherol, α-tocotrienol, β-tocopherol, and γ-tocopherol are statistically significant for the separation of the cluster groups.

**Figure 1:**
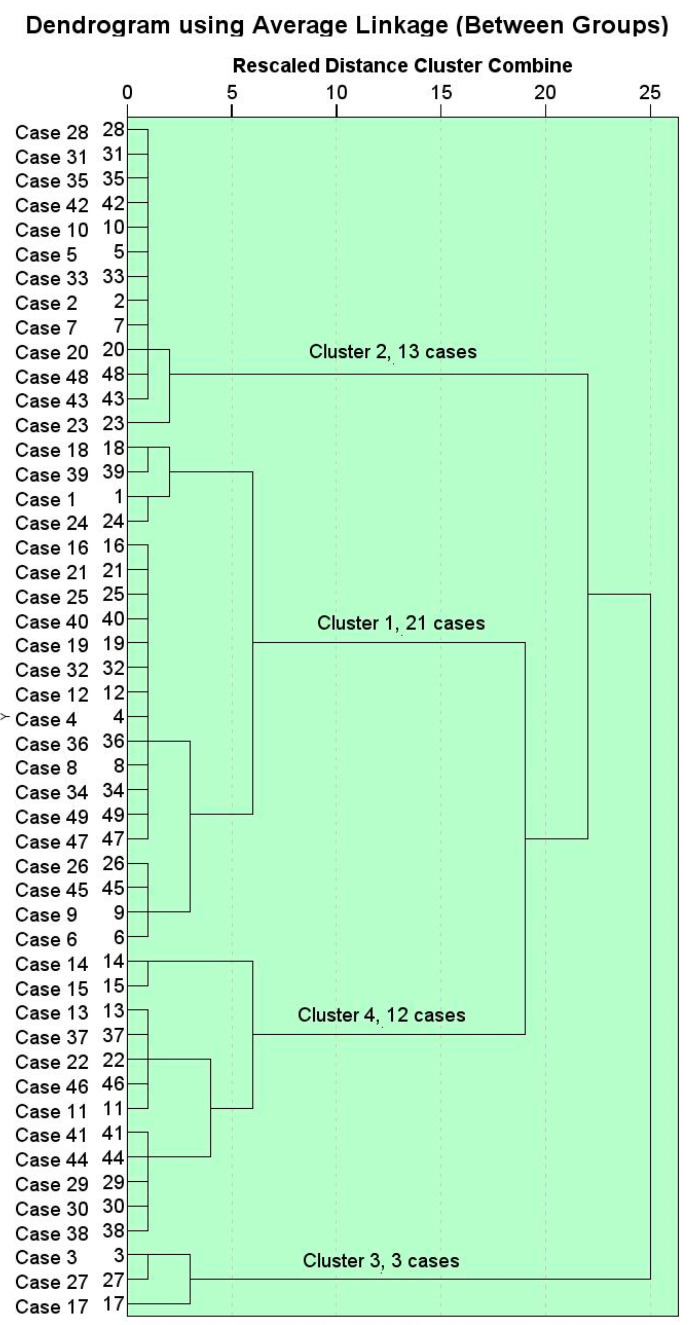
Dendrogram from a hierarchical agglomerative algorithm with average-link clustering.

The plants were clearly grouped into four cluster groups, as seen in the dendrogram in Figure 1.

The distances between the clusters are at least 10 rescaled cluster distances. For the resulting clustering, the Davies–Bouldin index was calculated, which measures the compactness of the clusters (how close the points in the cluster are to each other) and their separation (how different each cluster is from the others). The index value of 0.80 indicates well-defined clusters. The silhouette score was calculated for the data, and its value is 0.55. This indicates a moderate clustering quality, meaning that the clusters are reasonably well-formed. A score of 0.55 suggests that most data points are closer to their assigned cluster than to other clusters.

The first cluster includes 21 plants: *A. sardoum*, *A. officinalis*, *B. trigyna*, *S. ruthenica*, *C. creticus*, *B. cretica*, *C. laevigata*, *E. helioscopia*, *G. biloba*, *J. compressus*, *L. salicaria*, *A. heldreichii*, *A. officinalis*, *R. luteola*, *P. spina-christi*, *R. graveolens*, *V. lychnitis*, *A. altissima*, *C. australis*, *V. agnus-castus*, and *Z. fabago.* In the second cluster, 13 plants are grouped: *C. coggygria*, *N. pallens*, *C. album*, *C. sepium*, *E. ciconium*, *V. album*, *B. papyrifera*, *P. oleracea*, *R. lutea*, *H. suaveolens*, *N. tabacum*, *S. erectum*, and *V. arvensis.* In the third cluster, there are only three plants: *R. typhina*, *C. transsilvanica*, and *M. silvestris*, and in the fourth there are 12, as follows: *B. alba*, *C. monogyna*, *C. esculentus*, *D. communis*, *A. lutea*, *O. biennis*, *P. europaea*, *K. paniculata*, *D. lanata*, *H. niger*, *T. natans*, and *U. dioica*.

As seen in Table 3, the main characteristic of the 21 plants grouped in the first cluster is the predominant presence of α-tocopherol, with an average of 63.733 units. In this cluster group, the average β-tocopherol content was the lowest, at 3.538 units. The second group of 13 plants is distinguished by the γ-tocopherol content, averaging 61.985 units. With an average of 0.915 units, this cluster stands out for having the lowest α-tocotrienol content. For the third group of only three plants, the distinguishing feature is the content of α-tocotrienol, with an average of 66.633 units. For this cluster group, the lowest content of γ-tocopherol is 4.267 units. For the fourth group of 12 plants, the significant characteristic is the content of β-tocopherol, with an average of 34.092 units, and the lowest content of α-tocotrienol at 1.133 units. In conclusion, we can say that clearly defined clustering identifies plants with high tocopherols and low tocotrienols in one group, or vice versa, with high tocotrienols and low tocopherols.

We used the advantages of the two-step cluster analysis, which combines the hierarchical method and the K-means method, to confirm the obtained cluster groups on the one hand, and to deduce the influence of the variables in the separation of the cluster groups on the other.

The two-step cluster analysis also identified four cluster groups. The resulting four cluster groups differ from those obtained with hierarchical clustering by the locations of only three elements grouped into other cluster groups, as shown in the cross-tabulation in Table 4. It can be seen from the table that two elements from the second cluster group and one from the fourth are grouped together with the elements from the first cluster in the two-step clustering.

The contingency coefficient between the two nominal variables indicating cluster membership is high (0.852) and statistically significant. We used the two-step clustering method to determine the influence of predictors in defining the cluster groups. Predictor Importance is calculated based on the contribution of each variable to the clustering, with values normalized between 0 and 1. The most important variable receives a value of 1, while the others are represented relative to it. The relative influence of the predictors is as follows: α-tocotrienol with a value of 1, β-tocopherol—0.941, γ-tocopherol—0.69, and α-tocopherol—0.304. This means that the division of cluster groups is most strongly influenced by α-tocotrienol and least influenced by α-tocopherol.

Based on the data reported, the tocopherol and tocotrienol composition in the seeds of the investigated plants differs even among plants belonging to the same botanical family. This implies that family membership is not an absolute indicator of the specific tocopherol composition of the plants. For example, plants of the same family, i.e., Malvaceae (e.g., *M. silvestris* and *A. officinalis*), are positioned in different clusters due to the considerable diversity in the composition of their oils. The result highlights that the tocopherol composition of the oils is probably linked more to biochemical adaptations and specific ecological conditions of the plants rather than to their taxonomy. For cluster patterns, the analysis indicates that particularly α-, β-, and γ-isomers are important predictors in cluster formation. Each cluster group is characterized by the dominance of a specific isomer of tocopherol or tocotrienol, which is statistically significant according to the ANOVA analysis.

On the other hand, cluster analysis allows plants to be grouped based on their tocopherol and tocotrienol composition, which reveals similarities between species that would appear to be distinct when botanical features are taken into account. The analysis demonstrates affinities between the composition of the plants that are not apparent from normal taxonomic groupings. This provides new perspectives for understanding plant biochemical adaptations. The grouping enables the selection of suitable plants for specific applications; for example, the production of oils rich in specific tocopherols that are appropriate for application in food, cosmetics, or pharmaceuticals. By the identification of plants with potential for industrial utilization, the research serves as a tool for the selection of valuable species for certain cultivation. Overall, cluster analysis provides structured information that facilitates both the understanding of plants’ biochemical characteristics and serves as a basis for identifying species with promising lipid-soluble antioxidant profiles for further exploration of their potential applications in various industrial sectors.

It should be noted that this preliminary study is based on composite samples, which integrate seeds collected from multiple individual plants at each site. This approach provides a representative biochemical snapshot of each species within its natural habitat but does not allow for the assessment of plant-to-plant variability within a site. Additionally, since all species were collected under natural field conditions, the observed variability likely reflects the combined effects of genetic differences and environmental influences. Future research involving controlled growth experiments, larger sample sizes, and site-specific replication across multiple years will be essential to fully disentangle these factors and to validate the patterns observed in this study.

## 3. Materials and Methods

### 3.1. Plant Materials

The study was conducted on 49 different plant species from 39 families, grown in the Plovdiv region of southern Bulgaria in 2022. Investigations were performed on air-dried seeds at technical ripeness. The plants were collected from three sites within the Plovdiv region. At least 30 individuals of each plant species were collected from each of the three sites. The region is a lowland (altitude between 169 and 182 m) with a temperate climate. Plants were harvested according to their natural ripening periods as follows: June–August (early ripening): Asparagaceae, Cistaceae, Geraniaceae, Liliaceae, Portulacaceae, and Violaceae; July–September (mid-season ripening): Alliaceae, Amaranthaceae, Boraginaceae, Chenopodiaceae, Cuscutaceae, Cyperaceae, Dipsacaceae, Juncaceae, Lythraceae, Malvaceae, Onagraceae, Plumbaginaceae, Resedaceae, Scrophulariaceae, Sparganiaceae, Urticaceae, and Zygophyllaceae; August–October (late ripening): Anacardiaceae, Convolvulaceae, Cucurbitaceae, Dioscoreaceae, Euphorbiaceae, Loranthaceae, Moraceae, Rhamnaceae, Rutaceae, Simaroubaceae, Solanaceae, Trapaceae, Ulmaceae, and Verbenaceae; September–October (late ripening): Ginkgoaceae and Sapindaceae. For each species, seeds were collected from natural populations at three sites within the region. Composite samples were then prepared by combining seeds from multiple individual plants within these populations. The climatic conditions were as follows: June 2022—the average temperatures were around 21 °C, total precipitation rate was 55 mm; July 2022—the average temperatures were 24 °C, total precipitation was 40 mm; August 2022—the average temperatures were 23 °C, total precipitation was 45 mm; September 2022—the average temperatures were 20 °C, total precipitation was 35 mm; and October 2022—the average temperatures were 14 °C, total precipitation was 35 mm.

### 3.2. Glyceride Oil Isolation

The oils were extracted using a Soxhlet apparatus with freshly distilled *n*-hexane for 8 h at 70 °C to prevent the degradation of thermolabile components. After the solvent’s rotation vacuum distillation, the extracted oils were dried under a stream of nitrogen and weighed [19].

### 3.3. Tocopherol Composition

Tocopherols and tocotrienols were analyzed directly in the oils by high-performance liquid chromatography (HPLC) with fluorescence detection [20]. A Merck-Hitachi unit fitted with column “Nucleosil” Si 50—5 250 mm × 4 mm (Merck, Darmstadt, Germany), fluorescent detector Merck-Hitachi F 1000 (Merck, Darmstadt, Germany), and D-2500 Merck-Hitachi chromato-integrator (Merck, Darmstadt, Germany) was used. The operating conditions were as follows: excitation at 295 nm, emission at 330 nm, mobile phase consisting of n-hexane and dioxane (96:4), column pressure of 50 bars, and a mobile phase flow rate of 1 cm^3^/min. The peaks were identified by using individual tocopherols and tocotrienols. Triplicate injections were performed with a 20 μL 2% solution in hexane for each oil.

The identification of the separate tocopherol and tocotrienol isomers in the investigated oils was determined by comparing the obtained data with those from solutions of pure α-, β-, γ-, and δ-tocopherols and tocotrienols (DL-α-, DL-β-, DL-γ, and DL-δ-tocopherols with purity ≥ 98%, Merck, Darmstadt, Germany, and Tocomin SupraBio, Carlson Laboratories, Inc., Arlington Heights, IL, USA). The quantification of the tocopherols in the samples was calculated using a calibration curve constructed by plotting the peak area (X-axis) against four different concentrations (Y-axis) for each tocopherol and tocotrienols—0.0125, 0.025, 0.05, and 0.1 mg/mL. A good linearity was obtained for the calibration curves of all components, with R^2^ ranging from 0.9730 (tocopherol) to 0.9976 (tocotrienol). The LOD (0.007–0.022 mg/mL) and LOQ (0.020–0.070 mg/mL) were also calculated. The results for the total tocopherols were expressed as mg/kg in the oils, and the individual tocopherol composition was given as a percentage of the total tocopherol content.

### 3.4. Description of the Data

Measurements were taken for the following ingredients in each plant: oil content, total tocopherol content (TTC), α-tocopherol, α-tocotrienol, β-tocopherol, β-tocotrienol, γ-tocopherol, γ-tocotrienol, δ-tocopherol, and δ-tocotrienol (in specific units of measurement). The descriptive statistics for the data are presented in Table 5.

**Figure 2:**
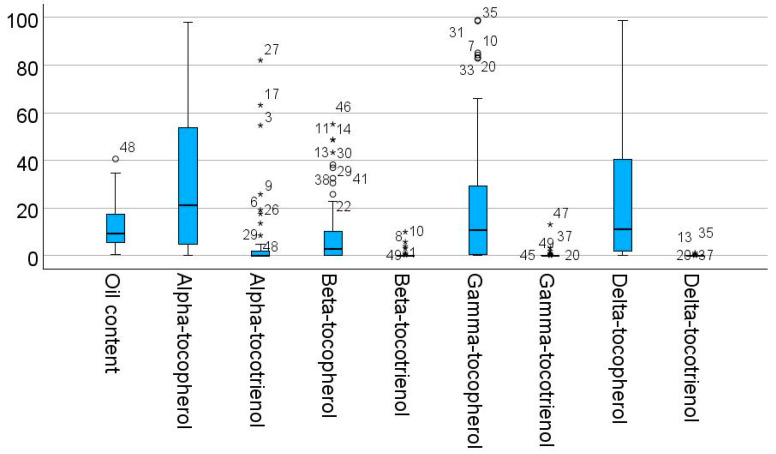
Box plot graphs of a subset of the studied variables.

The box plot graphs of some of the variables in Figure 2 show the presence of outliers, which shows that the selected set of plants has dissimilar content characteristics.

The aim of the present analysis is to group the plants into homogeneous clusters and identify the clustering characteristics that are fundamental for each group. For this purpose, we apply hierarchical cluster analysis.

### 3.5. Hierarchical Cluster Analysis

Cluster analysis is one of the unsupervised learning methods. By grouping data instances into subsets, clustering ensures that distinct instances belong to different groups while comparable instances are clustered together. The new groups are interesting in and of themselves, and their evaluation is intrinsic, as the objective of clustering is to identify a new set of categories. Formally, the clustering structure is represented as a set of subsets $S={⋃}_{i=1}^{k}C_{i}$, $C_{i}\subset S,$ such that $C_{i}\cap C_{j}=\emptyset$ for i≠j. Consequently, any instance in S belongs to exactly one and only one subset.

A basic technique for organizing data that is applied extensively in many scientific domains is hierarchical clustering. Agglomerative and divisive are the two basic categories into which hierarchical clustering can be generally divided [21,22,23]. One of the main benefits of this method is its capacity to reveal the data’s underlying structure by arranging items into a dendrogram, which is a hierarchical tree-like structure. This offers a visual and user-friendly method for investigating clusters at various granularities [24].

Selecting the right distance measure and linkage technique has a significant impact on hierarchical clustering success. Many clustering methods use distance measures (e.g., Euclidean distance, Manhattan distance, etc.) to determine the similarity or dissimilarity between any pair of objects.

In this study, we used average-link clustering (also called the minimum variance method) methods that consider the distance between two clusters to be equal to the average distance from any member of one cluster to any member of the other cluster [25]. Two-step cluster analysis is a hybrid clustering method that combines hierarchical clustering and K-means to efficiently group large datasets with both numerical and categorical variables. It automatically determines the optimal number of clusters using model selection criteria like the Bayesian information criterion (BIC) or the Akaike information criterion (AIC). The process consists of two steps: pre-clustering, where data points are grouped into small preliminary clusters, and hierarchical clustering, which merges these clusters into the final solution [26].

## 4. Conclusions

This study presents a detailed analytical characterization of glyceride oil content and tocopherol composition in seeds from 49 wild plant species representing 39 families of the Bulgarian flora. The observed variability in both oil yield and tocopherol profile underscores the substantial biochemical diversity among these species. High-performance liquid chromatography (HPLC) enabled the identification of distinct tocopherol and tocotrienol patterns, while hierarchical cluster analysis grouped the species into four major chemotypic clusters. The statistical significance of α-tocopherol, α-tocotrienol, β-tocopherol, and γ-tocopherol in differentiating these groups was confirmed via ANOVA.

Although the current work does not directly evaluate biological activity or industrial feasibility, the results provide a valuable reference point for identifying plant species with promising tocopherol profiles. These findings may provide a basis for future research focused on determining the functional properties, extraction efficiency, and application-specific potential of wild plant oils in nutritional, pharmaceutical, and cosmetic contexts. Ultimately, this study contributes to the foundational understanding of tocopherol diversity in underexplored wild species and supports ongoing efforts to identify novel natural antioxidant sources.

## Figures and Tables

**Table 1:** Content of glyceride oil in the seeds and tocopherols in the oils and seeds *.

Botanical Name (Family)	Oil Content	Content of Tocopherols, mg/kg	
	%	in the Oils	in the Seeds **
1. *Allium sardoum* Moris (Alliaceae)	1.2 ± 0.1 ^bc^	8066.5 ± 43.2 ^A^	96.8 ± 0.5 ^t^
2. *Cotinus coggygria* Scop. (Anacardiaceae)	8.4 ± 0.1 ^m^	1763.9 ± 26.7 ^x^	148.2 ± 2.2 ^v^
3. *Rhus typhina* L. (Anacardiaceae)	7.1 ± 0.2 ^jkl^	10.6 ± 1.3 ^abc^	0.8 ± 0.1 ^ab^
4. *Asparagus officinalis* L. (Asparagaceae)	11.9 ± 0.1 ^t^	214.9 ± 5.6 ^lm^	25.6 ± 0.7 ^l^
5. *Nonnea pallens* Petrovič (Boraginaceae)	18.1 ± 0.2 ^y^	21.9 ± 1.0 ^abc^	4.0 ± 0.2 ^bcd^
6. *Beta trigyna* Waldst. & Kit. (Chenopodiaceae)	1.7 ± 0.1 ^cd^	4.4 ± 0.5 ^a^	0.1 ± 0.0 ^a^
7. *Chenopodium album* L. (Amaranthaceae)	8.3 ± 0.1 ^m^	449.4 ± 8.2 ^p^	37.3 ± 0.7 ^n^
8. *Salsola ruthenica* Iljin (Chenopodiaceae)	10.5 ± 0.1 ^r^	178.5 ± 7.4 ^k^	18.8 ± 0.8 ^ij^
9. *Cistus creticus* L. (Cistaceae)	6.8 ± 0.1 ^j^	240.6 ± 10.6 ^mn^	13.4 ± 0.6 ^fg^
10. *Calystegia sepium* (L.) R. Br. (Convolvulaceae)	5.4 ± 0.1 ^hi^	829.2 ± 15.4 ^t^	44.8 ± 0.8 ^o^
11. *Bryonia alba* L. (Cucurbitaceae)	19.3 ± 0.3 ^z^	28.0 ± 2.4 ^abcd^	5.4 ± 0.5 ^cde^
12. *Bryonia cretica* L. (Cucurbitaceae)	17.5 ± 0.2 ^xy^	68.2 ± 4.3 ^fghi^	12.0 ± 0.8 ^fg^
13. *Cuscuta monogyna* Wahl. (Cuscutaceae)	5.6 ± 0.1 ^i^	2858.2 ± 25.3 ^y^	162.7 ± 1.4 ^x^
14. *Cyperus esculentus* L. (Cyperaceae)	22.5 ± 0.2 ^B^	134.0 ± 6.7 ^j^	30.2 ± 1.5 ^m^
15. *Dioscorea communis* (L.) Caddick & Wilkin (Dioscaraceae)	9.4 ± 0.1 ^op^	187.2 ± 5.4 ^kl^	17.6 ± 0.5 ^hi^
16. *Cephalaria laevigata* W.K.Schrad. (Dipsaceae)	18.8 ± 0.1 ^z^	79.9 ± 4.8 ^hi^	15.1 ± 0.9 ^gh^
17. *Cephalaria transsilvanica* Schrad. (Dipsaceae)	21.1 ± 0.2 ^A^	31.5 ± 2.5 ^abcde^	6.6 ± 0.5 ^de^
18. *Euphorbia helioscopia* L. (Euphorbiaceae)	2.2 ± 0.1 ^de^	41.6 ± 3.7 ^bcdefg^	0.9 ± 0.1 ^ab^
19. *Ginkgo biloba* L. (Ginkgoaceae)	4.2 ± 0.1 ^f^	58.3 ± 5.2 ^defghi^	2.4 ± 0.2 ^abc^
20. *Erodium cicutarium* (L.) L’Hér. (Geraniaceae)	12.0 ± 0.1 ^t^	928.3 ± 10.4 ^u^	111.4 ± 1.2 ^u^
21. *Juncus compressus* Jacq. (Juncaceae)	1.2 ± 0.1 ^bc^	564.3 ± 12.1 ^q^	6.8 ± 0.1 ^de^
22. *Asphodeline lutea* Rchb. (Liliaceae)	21.4 ± 0.2 ^A^	233.1 ± 6.4 ^mn^	49.9 ± 1.4 ^p^
23. *Viscum album* L. (Santalaceae)	7.4 ± 0.2 ^kl^	1077.4 ± 16.7 ^v^	79.8 ± 1.2 ^rs^
24. *Lythrum salicaria* L. (Lythraceae)	13.4 ± 0.1 ^u^	88.0 ± 4.1 ^i^	11.8 ± 0.5 ^f^
25. *Althaea heldreichii* Boiss. (Malvaceae)	9.0 ± 0.1 ^no^	35.8 ± 1.6 ^abcdef^	3.2 ± 0.1 ^abc^
26. *Althaea officinalis* L. (Malvaceae)	1.4 ± 0.1 ^c^	12.8 ± 1.2 ^abc^	0.2 ± 0.0 ^a^
27. *Malva silvestris* L. (Malvaceae)	2.6 ± 0.1 ^e^	47.6 ± 1.8 ^cdefgh^	1.2 ± 0.1 ^ab^
28. *Broussonetia papyrifera* (L.) Vent. (Moraceae)	26.0 ± 0.4 ^D^	1494.8 ± 14.2 ^w^	388.3 ± 3.7 ^z^
29. *Oenothera biennis* L. (Onagraceae)	11.2 ± 0.2 ^s^	64.8 ± 3.6 ^efghi^	7.3 ± 0.4 ^e^
30. *Plumbago europaea* L. (Plumbaginaceae)	4.9 ± 0.2 ^gh^	32.8 ± 2.1 ^abcdef^	1.6 ± 0.1 ^ab^
31. *Portulaca oleracea* L. (Portulaceae)	8.7 ± 0.2 ^mn^	253.4 ± 4.6 ^n^	22.0 ± 0.4 ^jk^
32. *Reseda luteola* L. (Resedaceae)	7.5 ± 0.1 ^l^	13.9 ± 1.4 ^abc^	1.0 ± 0.1 ^ab^
33. *Reseda lutea* L. (Resedaceae)	15.5 ± 0.2 ^w^	35.0 ± 0.9 ^abcdef^	5.4 ± 0.1 ^cde^
34. *Paliurus spina-christi* Mill. (Rhamnaceae)	17.0 ± 0.2 ^x^	236.0 ± 3.4 ^mn^	40.1 ± 0.6 ^n^
35. *Haplophyllum suaveolens* (DC.) G.Don (Rutaceae)	11.2 ± 0.2 ^s^	632.7 ± 7.8 ^r^	71.1 ± 0.9 ^q^
36. *Ruta graveolens* L. (Rutaceae)	14.6 ± 0.1 ^v^	174.0 ± 2.4 ^k^	25.4 ± 0.4 ^l^
37. *Koelreuteria paniculata* Laxm. (Sapindaceae)	7.5 ± 0.1 ^l^	1099.0 ± 8.6 ^v^	82.6 ± 0.6 ^s^
38. *Digitalis lanata* Ehrh. (Scrophulariaceae)	9.8 ± 0.2 ^p^	241.1 ± 3.3 ^mn^	23.6 ± 0.3 ^kl^
39. *Verbascum lychnitis* L. (Scrophulariaceae)	5.7 ± 0.1 ^i^	3378.3 ± 18.6 ^z^	193.0 ± 1.0 ^y^
40. *Ailanthus altissima* (Mill.) Swingle (Simarubaceae)	17.0 ± 0.3 ^x^	76.3 ± 3.5 ^ghi^	13.0 ± 0.6 ^fg^
41. *Hyoscyamus niger* L. (Solanaceae)	23.4 ± 0.2 ^C^	9.2 ± 0.8 ^ab^	2.2 ± 0.2 ^abc^
42. *Nicotiana tabacum* L. (Solanaceae)	34.5 ± 0.4 ^E^	224.3 ± 5.4 ^mn^	77.6 ± 1.9 ^r^
43. *Sparganium erectum* L. (Sparganiaceae)	0.7 ± 0.0 ^ab^	239.6 ± 6.1 ^mn^	1.7 ± 0.1 ^ab^
44. *Trapa natans* L. (Trapaceae)	0.5 ± 0.0 ^a^	91.2 ± 2.8 ^i^	0.5 ± 0.0 ^a^
45. *Celtis australis* L. (Ulmaceae)	21.0 ± 0.2 ^A^	727.6 ± 8.1 ^s^	152.7 ± 1.7 ^w^
46. *Urtica dioica* L. (Urticaceae)	22.7 ± 0.1 ^B^	163.8 ± 2.6 ^jk^	37.3 ± 0.6 ^n^
47. *Vitex agnus-castus* L. (Verbenaceae)	4.5 ± 0.1 ^fg^	18.2 ± 0.5 ^abc^	0.8 ± 0.0 ^ab^
48. *Viola arvensis* Murray (Violaceae)	40.6 ± 0.2 ^F^	234.5 ± 2.8 ^mn^	95.5 ± 1.1 ^t^
49. *Zygophyllum fabago* L. (Zygophyllaceae)	6.9 ± 0.1 ^jk^	357.8 ± 6.4 ^o^	24.7 ± 0.4 ^kl^

*—The results are mean ± standard deviation (*n* = 3); Different letters represent significant difference among the results in a column (*p* < 0.05, Tukey HSD test); **—The total tocopherol content in the seeds was calculated from the tocopherol content determined in the oil, based on the sample’s glyceride oil content.

**Table 2:** Individual tocopherol composition of the glyceride oils (% of total tocopherol content) *.

Botanical Name	α **	α-3	β	β-3	γ	γ-3	δ	δ-3
1. *Allium sardoum* Moris	47.2 ± 0.2	0.2 ± 0.0	1.2 ± 0.1	1.2 ± 0.0	48.4 ± 0.3	n.d. ***	1.8 ± 0.1	n.d.
2. *Cotinus coggygria* Scop.	0.3 ± 0.0	n.d.	2.5 ± 0.1	n.d.	16.3 ± 0.1	1.1 ± 0.1	79.8 ± 0.4	n.d.
3. *Rhus typhina* L.	10.0 ± 0.2	54.7 ± 0.2	17.0 ± 0.1	n.d.	7.0 ± 0.1	n.d.	11.0 ± 0.2	n.d.
4. *Asparagus officinalis* L.	51.2 ± 0.2	4.8 ± 0.1	3.3 ± 0.1	n.d.	n.d.	n.d.	40.6 ± 0.3	n.d.
5. *Nonnea pallens* Petrovič	n.d.	n.d.	3.6 ± 0.1	n.d.	59.1 ± 0.2	n.d.	37.3 ± 0.1	n.d.
6. *Beta trigyna* Waldst. & Kit.	29.7 ± 0.2	17.6 ± 0.1	n.d.	n.d.	n.d.	n.d.	52.7 ± 0.2	n.d.
7. *Chenopodium album* L.	16.8 ± 0.1	n.d.	n.d.	n.d.	83.2 ± 0.1	n.d.	n.d.	n.d.
8. *Salsola ruthenica* Iljin.	79.1 ± 0.2	n.d.	5.2 ± 0.1	0.6 ± 0.0	13.0 ± 0.2	0.6 ± 0.0	1.4 ± 0.1	n.d.
9. *Cistus creticus* L.	66.2 ± 0.3	25.8 ± 0.2	0.2 ± 0.0	n.d.	7.8 ± 0.1	n.d.	n.d.	n.d.
10. *Calystegia sepium* (L.) R. Br.	5.0 ± 0.1	0.1 ± 0.0	2.9 ± 0.1	3.1 ± 0.1	85.1 ± 0.5	n.d.	3.8 ± 0.1	n.d.
11. *Bryonia alba* L.	16.6 ± 0.1	n.d.	48.7 ± 0.2	n.d.	13.7 ± 0.2	n.d.	21.0 ± 0.2	n.d.
12. *Bryonia cretica* L.	85.5 ± 0.4	n.d.	5.2 ± 0.1	n.d.	7.3 ± 02	n.d.	1.9 ± 0.1	n.d.
13. *Cuscuta monogyna* Wahl.	4.9 ± 0.1	n.d.	43.3 ± 0.1	n.d.	0.3 ± 0.0	n.d.	50.3 ± 0.2	1.2 ± 0.1
14. *Cyperus esculentus* L.	50.1 ± 0.4	n.d.	48.6 ± 0.2	n.d.	1.2 ± 0.1	n.d.	0.1 ± 0.0	n.d.
15. *Dioscorea communis* (L.) Caddick & Wilkin	4.1 ± 0.1	n.d.	4.2 ± 0.1	n.d.	n.d.	n.d.	91.7 ± 0.3	n.d.
16. *Cephalaria laevigata* W.K.Schrad.	81.1 ± 0.2	3.9 ± 0.1	2.9 ± 0.1	3.9 ± 0.1	5.7 ± 0.2	n.d.	2.5 ± 0.1	n.d.
17. *Cephalaria transsilvanica* Schrad.	31.0 ± 0.2	63.2 ± 0.4	n.d.	n.d.	5.8 ± 0.1	n.d.	n.d.	n.d.
18. *Euphorbia helioscopia* L.	36.1 ± 0.2	2.0 ± 0.0	1.4 ± 0.1	n.d.	21.4 ± 0.1	n.d.	39.1 ± 0.2	n.d.
19. *Ginkgo biloba* L.	76.7 ± 0.2	n.d.	6.9 ± 0.1	n.d.	3.4 ± 0.1	n.d.	13.0 ± 0.1	n.d.
20. *Erodium cicutarium* (L.) L’Hér.	12.7 ± 0.1	n.d.	1.4 ± 0.1	n.d.	84.2 ± 0.3	0.2 ± 0.0	1.4 ± 0.1	0.1 ± 0.0
21. *Juncus compressus* Jacq.	91.1 ± 0.4	n.d.	1.7 ± 0.2	n.d.	3.1 ± 0.1	n.d.	4.1 ± 0.1	n.d.
22. *Asphodeline lutea* Rchb.	0.9 ± 0.0	n.d.	25.8 ± 0.2	n.d.	0.5 ± 0.0	n.d.	72.8 ± 0.4	n.d.
23. *Viscum album* L.	27.5 ± 0.2	n.d.	n.d.	n.d.	66.0 ± 0.2	3.2 ± 0.1	3.3 ± 0.1	n.d.
24. *Lythrum salicaria* L.	46.4 ± 0.2	n.d.	8.8 ± 0.1	n.d.	36.7 ± 0.2	n.d.	8.0 ± 0.2	n.d.
25. *Althaea heldreichii* Boiss.	98.0 ± 0.5	2.0 ± 0.1	n.d.	n.d.	n.d.	n.d.	n.d.	n.d.
26. *Althaea officinalis* L.	53.7 ± 0.3	19.2 ± 0.1	n.d.	n.d.	n.d.	n.d.	27.1 ± 0.2	n.d.
27. *Malva silvestris* L.	8.3 ± 0.1	82.0 ± 0.2	n.d.	n.d.	n.d.	n.d.	9.7 ± 0.2	n.d.
28. *Broussonetia papyrifera* (L.) Vent.	0.4 ± 0.0	n.d.	n.d.	n.d.	25.5 ± 0.2	n.d.	74.1 ± 0.2	n.d.
29. *Oenothera* biennis L.	21.3 ± 0.2	13.6 ± 0.2	30.5 ± 0.1	n.d.	23.1 ± 0.1	n.d.	11.5 ± 0.1	n.d.
30. *Plumbago europaea* L.	20.3 ± 0.1	n.d.	38.2 ± 0.2	n.d.	25.5 ± 0.2	n.d.	16.0 ± 0.1	n.d.
31. *Portulaca oleracea* L.	1.0 ± 0.1	n.d.	n.d.	n.d.	98.8 ± 0.1	n.d.	0.2 ± 0.0	n.d.
32. *Reseda luteola* L.	74.7 ± 0.3	n.d.	9.1 ± 0.1	n.d.	5.7 ± 0.1	n.d.	10.5 ± 0.1	n.d.
33. *Reseda lutea* L.	6.9 ± 0.1	n.d.	n.d.	n.d.	82.8 ± 0.2	n.d.	10.3 ± 0.1	n.d.
34. *Paliurus spina-christi* Mill.	87.7 ± 0.4	n.d.	n.d.	n.d.	10.8 ± 0.2	n.d.	1.5 ± 0.1	n.d.
35. *Haplophyllum suaveolens* (DC.) G.Don	0.2 ± 0.0	n.d.	n.d.	n.d.	98.5 ± 0.2	n.d.	0.6 ± 0.0	0.7 ± 0.1
36. *Ruta graveolens* L.	52.9 ± 0.2	n.d.	8.5 ± 0.1	5.8 ± 0.1	0.5 ± 0.0	2.0 ± 0.1	30.3 ± 0.3	n.d.
37. *Koelreuteria paniculata* Laxm.	2.1 ± 0.1	n.d.	22.5 ± 0.2	n.d.	n.d.	0.2 ± 0.0	75.0 ± 0.4	0.3 ± 0.0
38. *Digitalis lanata* Ehrh.	23.7 ± 0.2	n.d.	36.9 ± 0.2	n.d.	24.0 ± 0.1	n.d.	15.4 ± 0.1	n.d.
39. *Verbascum lychnitis* L.	64.3 ± 0.2	0.1 ± 0.0	3.4 ± 0.1	n.d.	29.2 ± 0.2	0.6 ± 0.0	2.4 ± 0.1	n.d.
40. *Ailanthus altissima* (Mill.) Swingle	96.4 ± 0.5	n.d.	3.6 ± 0.2	n.d.	n.d.	n.d.	n.d.	n.d.
41. *Hyoscyamus niger* L.	21.1 ± 0.1	n.d.	32.5 ± 0.2	n.d.	14.8 ± 0.1	n.d.	31.6 ± 0.2	n.d.
42. *Nicotiana tabacum* L.	1.6 ± 0.1	n.d.	1.0 ± 0.0	n.d.	52.0 ± 0.3	n.d.	45.4 ± 0.2	n.d.
43. *Sparganium erectum* L.	1.9 ± 0.1	3.3 ± 0.1	2.9 ± 0.1	n.d.	7.3 ± 0.1	n.d.	84.6 ± 0.2	n.d.
44. *Trapa natans* L.	13.7 ± 0.2	n.d.	22.7 ± 0.2	n.d.	8.2 ± 0.1	n.d.	55.4 ± 0.2	n.d.
45. *Celtis australis* L.	0.7 ± 0.1	0.3 ± 0.0	n.d.	n.d.	n.d.	0.3 ± 0.0	98.7 ± 0.2	n.d.
46. *Urtica dioica* L.	0.5 ± 0.0	n.d.	55.2 ± 0.3	n.d.	n.d.	n.d.	44.3 ± 0.2	n.d.
47. *Vitex agnus-castus* L.	42.2 ± 0.2	2.3 ± 0.1	10.3 ± 0.1	10.0 ± 0.1	13.3 ± 0.1	13.1 ± 0.1	8.8 ± 0.1	n.d.
48. *Viola arvensis* Murray	12.4 ± 0.2	8.5 ± 0.1	n.d.	n.d.	47.0 ± 0.2	n.d.	32.1 ± 0.2	n.d.
49. *Zygophyllum fabago* L.	77.5 ± 0.4	n.d.	2.6 ± 0.1	0.3 ± 0.0	19.3 ± 0.1	0.3 ± 0.0	n.d.	n.d.

* The results are mean ± standard deviation (*n* = 3); ** α—α-tocopherol, α-3—α-tocotrienol, β—β-tocopherol, β-3—β-tocotrienol, γ—γ-tocopherol, γ-3—γ-tocotrienol, δ—δ-tocopherol, δ-3—δ-tocotrienol; *** n.d.—Not detected.

**Table 3:** Descriptive statistics of the four clusters.

Cluster	Statistics	α-Tocopherol	α-Tocotrienol	β-Tocopherol	γ-Tocopherol
1	Mean	63.733	3.724	3.538	10.743
	N	21	21	21	21
	Std. Deviation	24.8678	7.4314	3.4228	13.4388
2	Mean	6.669	0.915	1.100	61.985
	N	13	13	13	13
	Std. Deviation	8.4429	2.4539	1.3922	30.7642
3	Mean	16.433	66.633	5.667	4.267
	N	3	3	3	3
	Std. Deviation	12.6437	13.9701	9.8150	3.7434
4	Mean	14.942	1.133	34.092	9.275
	N	12	12	12	12
	Std. Deviation	14.1845	3.9260	14.2090	10.4590
Total	Mean	33.749	6.196	10.504	23.582
	N	49	49	49	49
	Std. Deviation	32.0527	16.7662	15.5366	29.7243

**Table 4:** Crosstab between the clusters obtained from average-link clustering and two-step clustering.

Count		Two-Step Cluster Number				
		1	2	3	4	Total
Average Linkage (Between Groups)	1	0	0	21	0	21
	2	0	11	2	0	13
	3	3	0	0	0	3
	4	0	0	1	11	12
Total		3	11	24	11	49

**Table 5:** Descriptive statistics of the studied variables.

Statistics	Mean	95% Confidence Interval for Mean		Median	Std. Deviation	Minimum	Maximum
		Lower Bound	Upper Bound				
Oil_content	11.833	9.293	14.372	9.4	8.8410121	0.5	40.6
TTC	572.498	201.392	943.604	178.5	1292.0017	4.4	8066.5
α-tocopherol	33.749	24.542	42.956	21.3	32.052659	0.0	98.0
α-tocotrienol	6.196	1.380	11.012	0.0	16.766211	0.0	82.0
β-tocopherol	10.504	6.041	14.967	2.9	15.536569	0.0	55.2
β-tocotrienol	0.508	0.004	1.012	0.0	1.7541423	0.0	10.0
γ-tocopherol	23.582	15.044	32.119	10.8	29.724335	0.0	98.8
γ-tocotrienol	23.582	15.044	32.119	10.8	29.724335	0.0	98.8
δ-tocopherol	24.959	16.658	33.260	11.0	28.900807	0.0	98.7
δ-tocotrienol	0.047	0.011	0.104	0.0	0.2001063	0.0	1.2

## Data Availability

The data presented in this study are available on request from the corresponding author.
